# Finsler fractional anisotropy (FFA): a directionally sensitive descriptor for multi-fiber white matter characterization in HARDI

**DOI:** 10.1038/s41598-026-48225-8

**Published:** 2026-04-14

**Authors:** Avinash Bansal, Sumit Kaushik, Jan Kubíček, Shalini Garg, Jan Slovák

**Affiliations:** 1 Department of Cybernetics and Biomedical Engineering, FEECS, VSB-Technical University of Ostrava, Ostrava, Poruba, Czech Republic; 2 Department of Mathematics, Masaryk University, Brno, Czech Republic

**Keywords:** Finsler metrics, White matter fibers, Anisotropy measure, Finsler fractional anisotropy, Fiber classification, Alzheimer’s disease, Biological techniques, Computational biology and bioinformatics, Engineering, Neurology, Neuroscience

## Abstract

Diffusion tensor imaging has significantly advanced the study of brain microstructure, but it faces challenges in regions with complex fiber architectures. High angular resolution diffusion imaging provides improved angular resolution, but still lacks straightforward scalar measures for complex diffusion patterns. We propose Finsler fractional anisotropy (FFA), a directionally sensitive biomarker derived from the Finsler geometry framework to capture local features in multi-fiber white matter. The descriptor was evaluated using both synthetic and in vivo data. Results suggest that FFA offers improved sensitivity in differentiating fiber crossings from parallel bundles, showing enhanced contrast and directional sensitivity, particularly within multi-fiber voxels where traditional scalar measures tend to be less informative. Statistical evaluations (t-test and Wilcoxon signed-rank test) indicated significant differences, with *p*-values $\ll 0.01$. We also discuss the strengths and limitations of FFA in analyzing complex white matter. Overall, these preliminary findings suggest that FFA may serve as a valuable complementary tool for characterizing complex brain microstructure. While results show enhanced directional sensitivity in regions with crossing fibers, further validation in larger clinical cohorts is required to establish its utility for investigating conditions such as multiple sclerosis and Alzheimer’s disease.

## Introduction

Diffusion magnetic resonance imaging (dMRI) has revolutionized the study of brain microstructure by enabling the non-invasive exploration of water diffusion patterns within biological tissues. By measuring the displacement of water molecules, this technique provides crucial insights into neural architecture and fiber pathways at the voxel level. However, accurate fiber reconstruction relies on precise diffusion characterization, particularly in regions with complex geometries such as fiber crossings, fanning, and merging fibers. Conventional diffusion models often struggle in these scenarios, potentially leading to misinterpretations of neural connectivity. Among the various dMRI frameworks, diffusion tensor imaging (DTI), introduced by Basser et al.^1,2^, remains one of the most widely applied techniques in clinical and research settings. The positive definite second-order tensor fundamental to DTI resides in Riemannian space, and extensive literature suggests that processing these tensors within this geometric framework is both mathematically efficient and physically sound^3–7^.

Second-order diffusion tensors provide several quantitative measures. True anisotropy descriptors include fractional anisotropy (FA), geodesic anisotropy (referred to as GeoA in recent studies to clearly distinguish it from generalized anisotropy, GA; previously denoted GA in earlier works), and Hilbert anisotropy (HA), which characterize the directional dependence of diffusion. FA, the most widely used scalar measure, summarizes the variance of the tensor’s eigenvalues relative to their mean^8^. GeoA quantifies anisotropy as the Riemannian (geodesic) distance between a tensor and its isotropic counterpart, making it invariant to scaling and rotation^9^. HA, in contrast, applies the Hilbert projective metric to the eigenvalues, producing a scale-invariant descriptor that reflects the contrast between the largest and smallest diffusivities. Although GeoA and HA are less common clinically, they offer mathematically rigorous alternatives to FA and may be advantageous in certain applications. Other related metrics, such as mean diffusivity (MD) and radial diffusivity (RD), quantify the average diffusivity and the diffusivity perpendicular to the principal direction, respectively, and are often reported alongside anisotropy measures^10^. Changes in these quantities can indicate alterations in water diffusion within pathological tissue, which is particularly important for examining diseases such as multiple sclerosis, Parkinson’s disease, and Alzheimer’s disease^11–17^.

DTI models water diffusion using a Gaussian framework represented by a second-order tensor. While diffusion is measured along multiple gradient directions (typically 6–30), the model collapses this data into a single predominant diffusion direction. This *single-tensor* assumption limits DTI’s ability to resolve complex fiber configurations, such as crossings, fanning, or merging, as it cannot represent multiple orientations within a single voxel. High angular resolution diffusion imaging (HARDI) addresses this by acquiring a denser sampling of the diffusion signal (typically 60–100 directions). Unlike DTI, HARDI is not constrained by Gaussian assumptions and employs advanced reconstruction techniques, such as Q-ball imaging, to estimate the orientation distribution function (ODF), thereby better capturing multiple fiber populations^18^.

In brain imaging, positive definite second-order tensors are commonly analyzed using Riemannian geometry, which helps capture white matter geometry. Nevertheless, modeling complex fiber geometries requires more advanced methods. Within the HARDI framework, higher-order tensors (HOTs) more effectively model orientation distribution functions (ODFs)^19,20^. Several anisotropy scalar measures derived from HOTs have been explored as biomarkers in clinical studies^21–24^. Examples include generalized fractional anisotropy (GFA)^21^, generalized anisotropy (GA)^22^, and multidirectional anisotropy (MDA)^23^. GFA extends traditional FA by incorporating higher-order diffusion models, better capturing complex diffusion profiles, especially in multi-fiber scenarios. GA focuses on overall variance in diffusion profiles, measuring deviation from isotropy across all directions. MDA analyzes anisotropy in multiple diffusion directions within a voxel, offering insights into fiber orientation.

Despite these advancements, higher-order tensor–based methods remain limited in regions with complex fiber crossings. Their shortcomings include: (i) underestimation of anisotropy, as multiple crossing fibers may appear isotropic even when individually anisotropic; (ii) inability to distinguish true isotropic diffusion from apparent isotropy caused by crossings; and (iii) scalar variations that often mimic FA, reducing their effectiveness in resolving multiple fiber populations^25,26^. These limitations arise because such measures collapse directional information into a single scalar, obscuring underlying microstructural complexity. An effective anisotropy measure should be sensitive to microstructural features such as axon density and myelination, robust against noise and partial volume effects, rotationally invariant, and capable of representing multiple fiber orientations. It should also provide biologically interpretable contrasts, distinguishing microscopic anisotropy intrinsic to fibers from macroscopic order reflecting overall alignment^27–29^.

To address some of these challenges, rotationally invariant descriptors derived from the Kelvin invariant formulation for 4th-order tensors have been proposed^30^. Computed using the Voigt-Mandel matrix^31,32^, eleven unique Kelvin invariants offer a rich set of contrasts. While these improve upon traditional approaches, they still struggle to fully capture fiber crossings, and their reliance on Riemannian metrics limits their representation of directional complexity. While GA, GFA, and Kelvin invariants provide overall anisotropy, they fail to capture detailed directional information in crossing regions.

To bridge this gap, we propose scalar measures based on the Finsler metric, which is more flexible and adaptive than Riemannian metrics. The Finsler framework adjusts to fiber geometry and diffusion profiles, capturing both directional and positional information. This offers potential for improved modeling of multi-fiber configurations in challenging regions such as fiber crossings. Preliminary applications of the Finsler metric in tractography using HARDI data have shown its potential advantages over traditional methods^33–36^. To the best of our knowledge, no established scalar biomarker has yet been developed within the Finsler framework. This gap motivates the introduction of Finsler fractional anisotropy (FFA) as a novel anisotropy descriptor.

Building on tractography applications, FFA is extended to fiber classification. It captures anisotropy across multiple orientations, providing more detailed classification of individual fibers and enhanced directional information in complex fiber intersections. FFA is evaluated on synthetic and in vivo datasets, showing that it can characterize up to three fibers per voxel and generally provides clearer separation compared to conventional descriptors. Based on its theoretical foundations and experimental results, FFA shows promise for advancing fiber modeling in research and may provide a useful framework for studying microstructural alterations in neurological diseases such as multiple sclerosis and Alzheimer’s disease.

The selection of the proposed descriptor is based on its promising theoretical foundations and preliminary results. However, exploring other potential descriptors could be valuable for a more comprehensive analysis.

## Results

This section evaluates the proposed descriptor and its stability to noise, demonstrating performance under different conditions. The assessment was conducted across varying noise levels, fiber configurations, and statistical tests, using both synthetic and in vivo data. Detailed results are presented below.

### Angular separation experiment

**Fig. 1:**
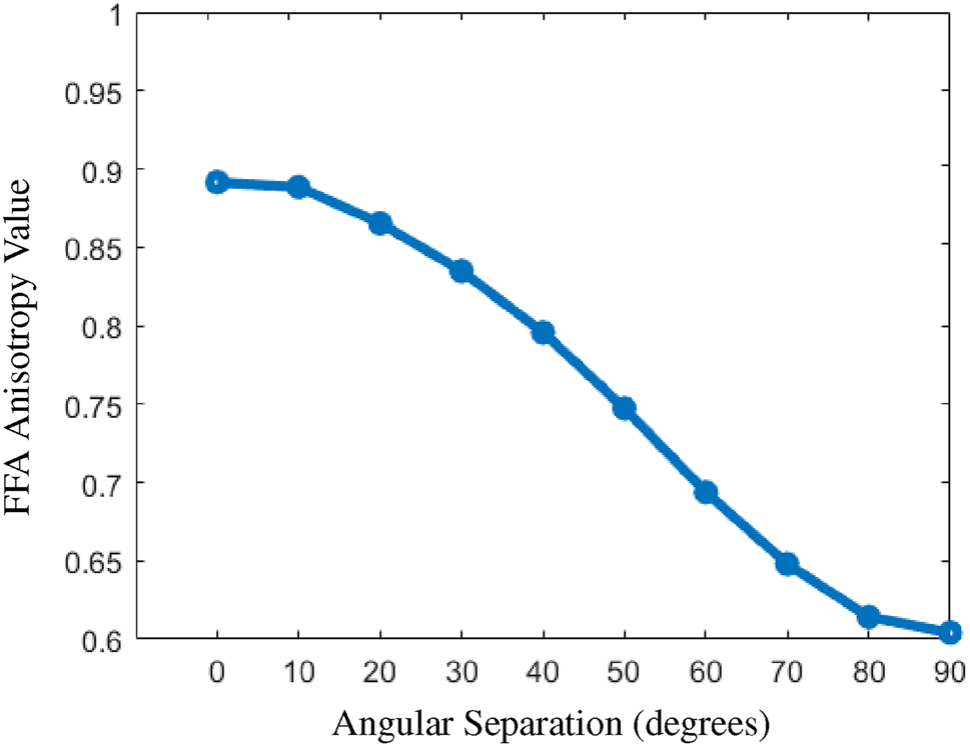
Line plot of FFA as a function of angular separation between two crossing fibers (synthetic data, no noise). FFA varies systematically with angular separation, reflecting the effect of fiber orientation on the anisotropy measure.

A two-fiber model was simulated within each voxel, with fibers rotated incrementally from $0^\circ$ to $90^\circ$ to represent a range of crossing angles. As shown in Fig. 1, FFA changes systematically with angular separation in this simulation. When the two fibers align in the same direction (equivalent to a single fiber), the anisotropy value approaches 1, consistent with aligned orientations.

The resulting FFA profile changes systematically with fiber crossing angles, particularly during transitions from near-parallel to orthogonal fibers. This behavior shows that FFA captures subtle variations in fiber arrangement, which may be useful for analyzing complex fiber structures.

### Tensor group simulations

Building on the angular separation experiment, four groups of tensors were simulated to represent different diffusion environments: isotropic diffusion (no fibers present), and voxels containing one, two, or three fiber populations. For the two- and three-fiber cases, the crossing angles were fixed at $90^\circ$. For synthetic datasets, FFA was computed by evaluating the direction-dependent Finsler metric over a uniform set of 81 unit directions on the sphere, using one representative per antipodal pair. For in vivo data, the same procedure was applied using the 64 available gradient directions. In both cases, voxelwise FFA was obtained by averaging the resulting Finsler metrics over the sampled directions, unless otherwise specified.

Consistency was tested by generating multiple instances of each group. To do this, fiber directions were rotated 50 times while maintaining the same crossing angle. Under practical conditions, small variations in FFA can occur due to numerical approximations, discretized gradient directions, and noise. This experiment, therefore, evaluated the stability of FFA under realistic conditions, showing separation of different diffusion patterns even in the presence of minor perturbations.

**Fig. 2:**
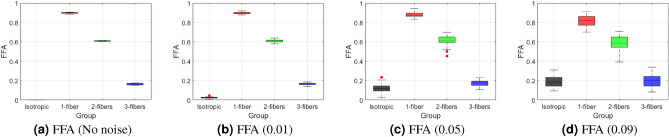
Box-and-whisker plots of FFA for separating diffusion environments at varying Rician noise levels. The vertical axis represents FFA anisotropy values for four groups: isotropic, single fiber, two orthogonal fibers, and three orthogonal fibers. From left to right, noise levels are: (**a**) no noise, (**b**) 0.01, (**c**) 0.05, and (**d**) 0.09. FFA shows sensitivity to noise, and at higher noise levels, the three-fiber case shows overlap with the isotropic voxel.

**Fig. 3:**
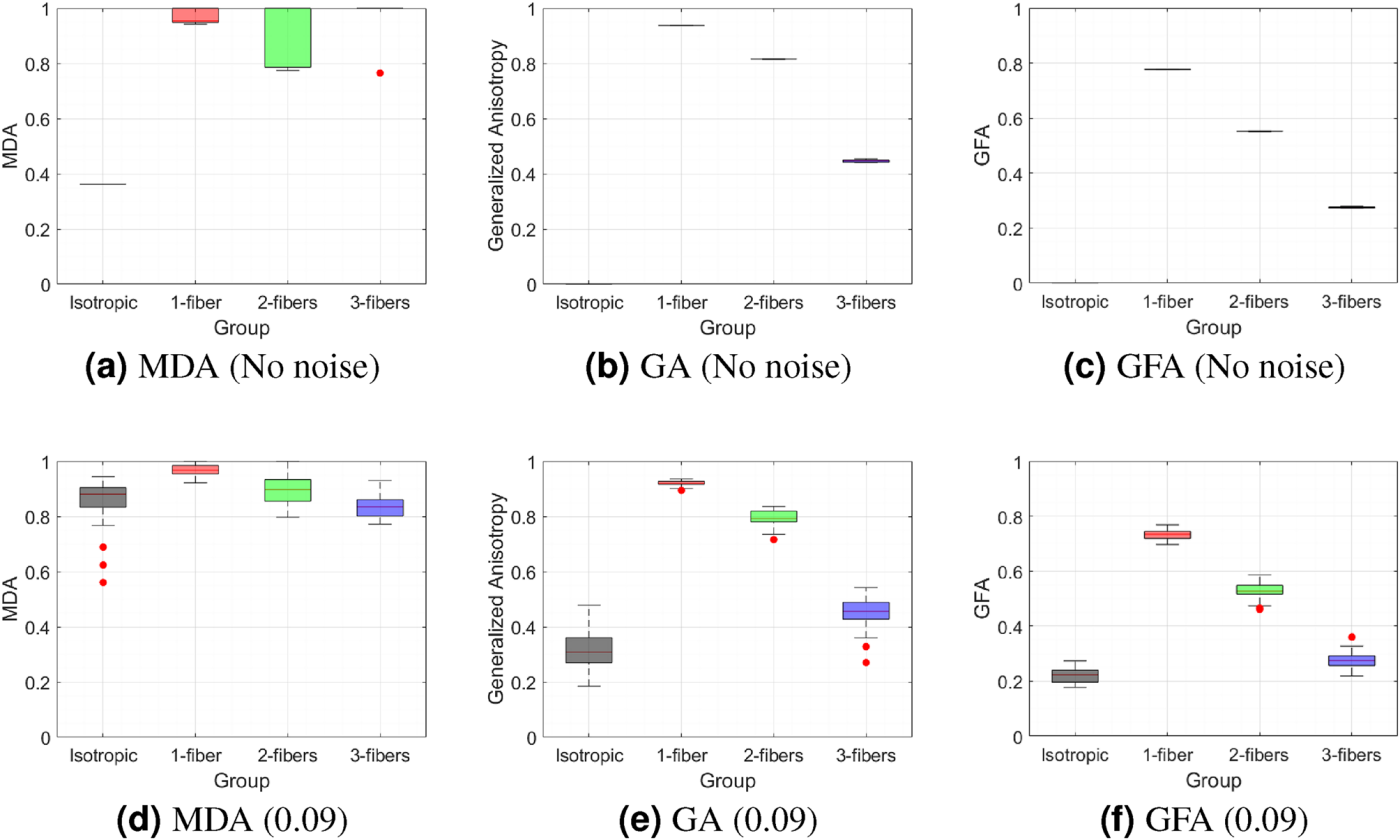
Box-and-whisker plot comparison of anisotropy measures: (**a**) Multidirectional anisotropy (MDA), (**b**) generalized anisotropy (GA), and (**c**) generalized fractional anisotropy (GFA) for various fiber configurations (isotropic, one fiber, two orthogonal fibers, and three orthogonal fibers). The top row shows performance without noise, and the bottom row shows the effect of Rician noise (0.09). The vertical axis represents the corresponding anisotropy values. These plots indicate that MDA is the least effective, while GA and GFA closely follow FFA and are more resilient to noise.

**Fig. 4:**
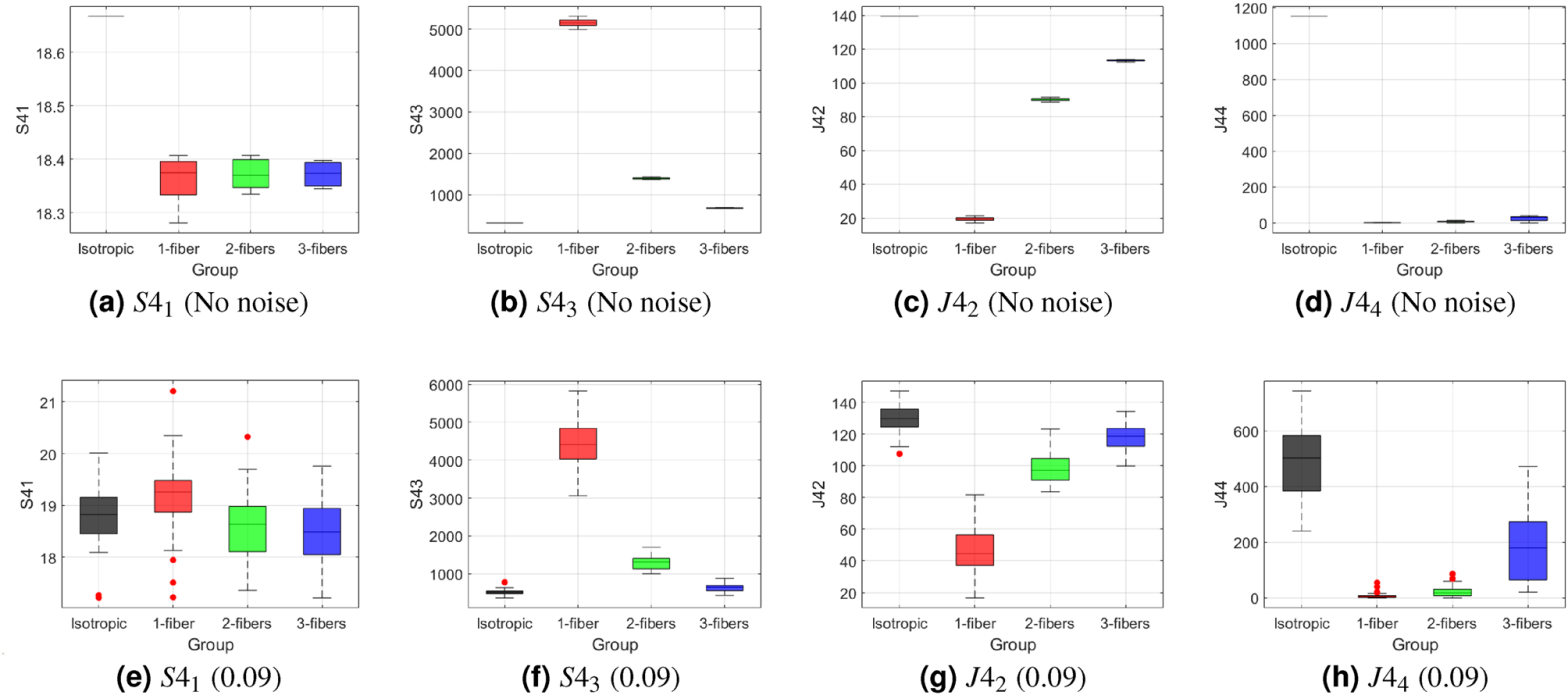
Box-and-whisker plots of selected Kelvin invariants: (**a**) $S4_1$, (**b**) $S4_3$, (**c**) $J4_2$, and (**d**) $J4_4$, for isotropic, single-fiber, two orthogonal fibers, and three orthogonal fibers. The top row shows results without noise, while the bottom row includes 0.09 Rician noise. The vertical axis represents the corresponding anisotropy values. These plots indicate that $S4_1$ and $J4_4$ are less effective, whereas $S4_3$ and $J4_2$ perform better but are more sensitive to noise.

Experiments were conducted at four Rician noise levels: 0.00 (noise-free), 0.01 (low), 0.05 (medium), and 0.09 (high). The corresponding signal-to-noise ratios (SNR) were infinity, 100, 20, and 11, respectively (see Table 4 in "Methods" section). Rician noise refers to random fluctuations in the measured signal that do not reflect actual changes in diffusion properties. These fluctuations introduce artificial variability into the measurements. Results are shown in Figs. 2, 3, and 4.

*We categorized the experiments as follows*

- *Noise-free (0.00) and Low-noise (0.01)* FFA values clearly separate the four groups (see Fig. 2a,b). The box-and-whisker plots show distinct interquartile ranges (IQRs), indicating FFA’s discriminative ability.
- *Medium-noise (0.05)* FFA still differentiates the four groups (see Fig. 2c), though variability increases. This shows that FFA retains sensitivity at moderate noise levels.
- *High-noise (0.09)* FFA shows overlap between the three-fiber and isotropic cases (see Fig. 2d), reducing classification accuracy. High noise increases measurement variability, limiting the effectiveness of FFA.

Overall, FFA effectively distinguishes between different fiber configurations in these simulations, but its sensitivity to noise should be considered when interpreting results.

### Comparison with existing anisotropy measures

To evaluate the performance of the proposed FFA descriptor, we compared it with several established, widely used anisotropy measures, including MDA, GA, GFA, and selected rotation-invariant Kelvin scalars. These comparisons are illustrated using box-and-whisker plots (see Figs. 3 and 4) to highlight the distinct behaviors of the scalars across different fiber configurations and noise conditions.

- *Multidirectional anisotropy (MDA)* This scalar (see Fig. 3a) fails to effectively distinguish and classify fiber configurations, even in the absence of noise. As noise increases (see Fig. 3d), it introduces additional confounding effects, further limiting its utility. Notably, MDA produces multiple values per voxel, allowing for various graphical representations. In general, MDA is defined for one of the many possible ODF extrema, rather than the global extremum across the entire ODF. Typically, the global ODF extremum is used for visualization along the most anisotropic direction. However, as demonstrated by^23^ in Figure 1 of their article, MDA was reported to be 0.82 for two orthogonal crossing fibers, highlighting its sensitivity to fiber complexity. While this suggests that MDA can capture certain anisotropic features, it fails to differentiate the four required regions, making it unsuitable for our analysis.
- *Generalized anisotropy (GA)* This scalar (see Fig. 3b) classifies fibers similarly to FFA in the absence of noise. However, when noise is introduced, GA exhibits less variation than FFA (see Fig. 3e). As demonstrated in^22^, GA incorporates a statistical multiplicative factor to enhance its classification performance by mapping the variance of diffusivities to a finite range. This transformation ensures a monotonic response to increasing anisotropy while maintaining numerical stability, making GA a useful metric for diffusion analysis. However, while GA appears effective in these examples due to orthogonal fiber crossings, its classification performance degrades as the fiber crossing angle decreases, as demonstrated in section "Evaluating descriptor performance on synthetic complex fiber arrangements".
- *Generalized fractional anisotropy (GFA)* This scalar (see Fig. 3c) classifies fibers similarly to FFA, with anisotropic values spanning a wide range. GFA is less sensitive to noise compared to FFA but still exhibits confounding effects when three fibers exhibit isotropic diffusion (see Fig. 3f). GFA provides a measure of overall diffusion anisotropy, similar to the initial purpose of FFA. While GFA gives a reliable scalar measure of anisotropy, it does not explicitly capture multidirectional information. In contrast, FFA derives its scalar values from a directionally adaptive framework, and details of its sensitivity to directional information are presented in Section "Directional sensitivity of FFA maps in synthetic data" and are further demonstrated on in vivo data in Section "Directional sensitivity of FFA maps in in vivo data"

All aforementioned anisotropy measures have their values confined to the range [0, 1].

*Comparison with Kelvin invariants* For rigorous testing, we further compared our results with rotation-invariant Kelvin scalars. We selected four scalars from a total of 11 different Kelvin invariants, based on their variability. These 4 were chosen to represent a balanced mix: some that performed best, some moderately well, and others that were least effective, providing a comprehensive comparison. It is worth noting that $S4_1$ corresponds to the mean diffusivity and does not provide anisotropy information; however, we included it here for completeness to illustrate the behavior of all Kelvin values. Box-and-whisker plots (see Fig. 4) reveal the distinct behaviors of these selected scalars.

- $S4_1$ and $J4_4$ These scalars (see Fig. 4a,d) were less effective in accurately characterizing fiber structures. Their limited ability to capture complex fiber configurations and subtle diffusion features reduced their utility for in-depth analysis. As a result, their performance was inadequate for detailed fiber analysis, making them unsuitable for our purposes.
- $S4_3$ and $J4_2$ These scalars (see Fig. 4b,c) exhibited performance similar to FFA. However, $J4_2$ introduced an inverted contrast. At higher noise levels, these scalars also exhibit overlapping groups, indicating reduced reliability under noisy conditions.

Unlike FFA, all Kelvin invariants have unbounded ranges, which limits their practical utility for reliable fiber characterization. Moreover, at higher noise levels (see Fig. 4e,f,g,h), these scalars struggled to accurately detect the number of fibers per voxel, emphasizing their sensitivity to noise compared to the stable and bounded behavior of FFA.

It is also important to note that we focused on FFA because of its observed ability to capture fiber configurations with bounded, intuitive contrast features, even in noisy conditions, which may support differentiation of fiber structures in detailed analysis.

### Statistical analysis

The effectiveness of FFA and its behavior under varying conditions were examined. Table 1 presents the mean and standard deviations of FFA, calculated across 50 different rotations for the four groups (as described previously). The low standard deviations suggest that the descriptor exhibits a high degree of rotational invariance, indicating that the descriptor varies little across the tested rotations and produces similar values across the tested fiber orientations. Such invariance is important for diffusion imaging analysis, as it allows the descriptor to represent the underlying fiber structures without being affected by orientation changes. The mean values indicate separation between the groups, suggesting sensitivity to different fiber configurations.

**Table 1:** Mean and standard deviation of FFA computed across 50 fiber rotations for four groups (isotropic, one fiber, two orthogonal fibers, and three orthogonal fibers per voxel), while preserving the constant angle between fibers. The values are obtained without noise.

Fiber group	FFA (Mean ± SD)
Isotropic	$0.00 \pm 0.000$
One fiber	$0.89 \pm 0.003$
Two orthogonal fibers	$0.59 \pm 0.002$
Three orthogonal fibers	$0.18 \pm 0.008$

In conjunction with these observations, Table 2 presents the results of t-tests and Wilcoxon signed-rank tests performed on the synthetic data for all possible pairwise comparisons between the four groups (isotropic, one fiber, two orthogonal fibers, and three orthogonal fibers). The inclusion of both tests was deliberate: the t-test evaluates differences under the assumption of normality and homogeneity of variances, whereas the Wilcoxon signed-rank test is a non-parametric alternative that does not rely on these assumptions and is less affected by outliers. The low* p*-values ($p \ll 0.01$) observed in nearly all tests with Rician noise levels $\le 0.05$ indicate statistically significant differences between the groups in terms of FFA. These findings suggest that the descriptor can separate groups, even under moderate noise conditions. Using both tests supports the consistency of the results across different statistical assumptions.

**Table 2:** FFA results of the t-test and Wilcoxon signed-rank test under different Rician noise conditions, showing p-values for the six possible pairs of features.

Rician noise	$\le {0.05}$		0.09	
Possible pairs	t-test	Wilcoxon	t-test	Wilcoxon
Isotropic vs. One fiber	—	—	—	—
Isotropic vs. Two orthogonal fibers	—	—	—	—
Isotropic vs. Three orthogonal fibers	—	—	0.71	—
One fiber vs. Two orthogonal fibers	—	—	—	—
One fiber vs. Three orthogonal fibers	—	—	—	—
Two orthogonal fibers vs. Three orthogonal fibers	—	—	—	—

Low *p*-values indicate significant statistical differences between the distributions. Here “**—**” represents p $\ll 0.01$, indicating highly significant differences.

*Note* Rotation invariance was verified to minimize implementation errors and identify potential non-invariant artifacts such as discretization. The results in Table 1 indicate this invariance under the tested conditions. Table 2 shows the t-test and Wilcoxon signed-rank test results, indicating significant differences between most fiber configurations, even under noise. While the current setup uses an *in-silico* phantom with fixed orthogonal crossings, the descriptor separates fiber groups. Further investigation with varied crossing angles and fiber properties will help assess its performance in more realistic scenarios.

### Stability and regularization studies of FFA

This section examines the stability of FFA across different diffusion environments, directional sampling densities, and noise conditions. For these experiments, synthetic voxels representing isotropic diffusion, single fiber, two orthogonal fibers, and three orthogonal fibers were generated as described in the previous section. FFA values were computed using the same direction sampling and averaging procedure while increasing the number of uniformly distributed unit directions on the sphere. The number of gradient directions per voxel (*N*) was varied from 50 to 3000. Experiments were repeated across 30 independent realizations for each direction set and variable noise level to enable quantitative assessment of convergence, variability, and stability of FFA.

#### Stability of FFA under directional sampling and rician noise

**Fig. 5:**
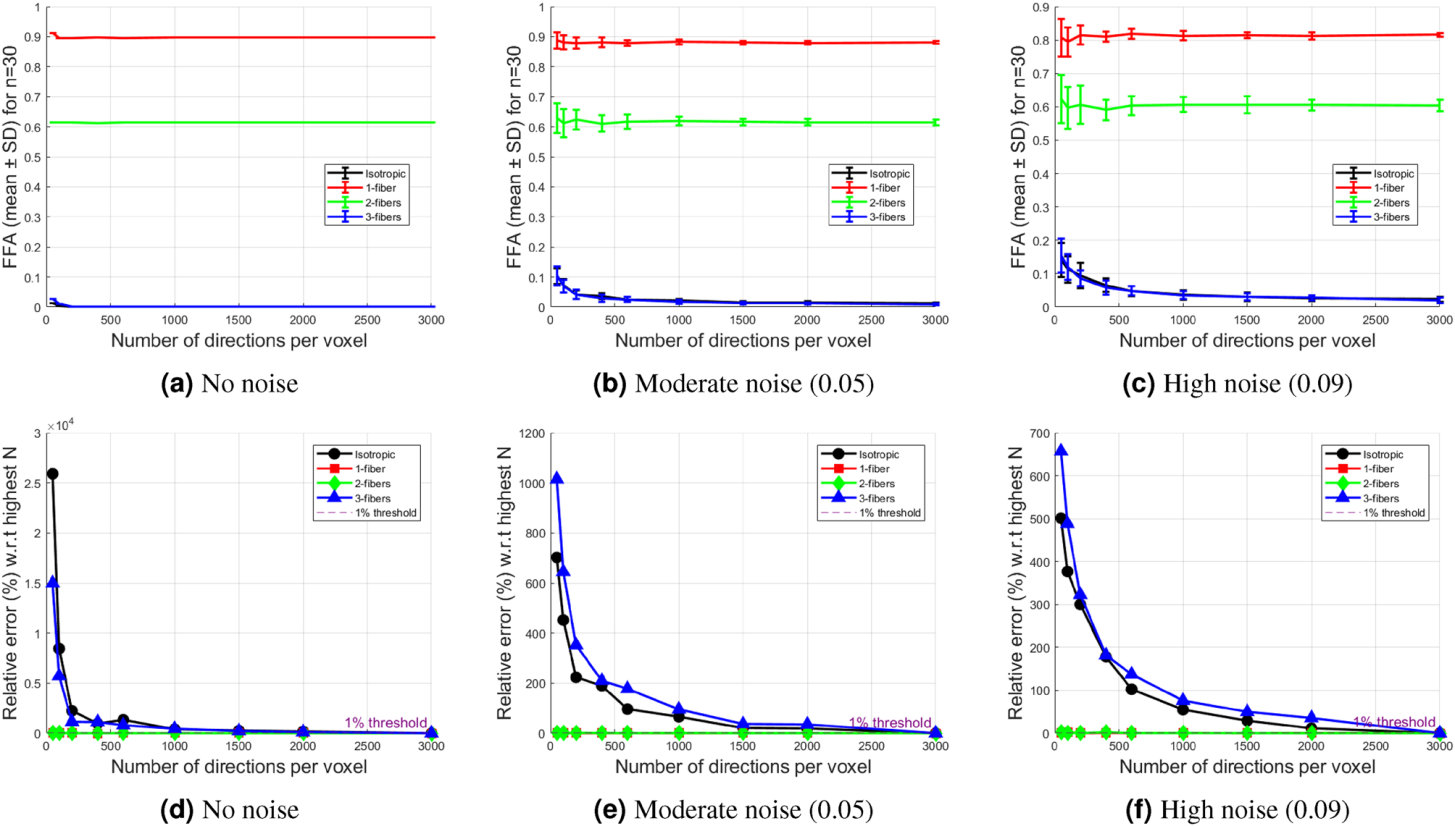
FFA stability and convergence with increasing directional sampling density under different Rician noise levels. (**a**–**c**) Mean FFA ± SD across 30 independent realizations versus the number of directions per voxel for (**a**) no noise, (**b**) 0.05 Rician noise, (**c**) 0.09 Rician noise. (**d**–**f**) Relative error (%) with respect to the high-density reference ($N=3000$) for the same noise levels, with a 1% threshold line. FFA stabilizes rapidly for 1-fiber and 2-fiber configurations, and more gradually for isotropic and 3-fiber configurations, with SD narrowing and relative error dropping below 1% for $N \ge 1500$–2000 in the latter cases.

Figure 5 illustrates the stability of FFA for the simulated voxel configurations described above as the number of gradient directions per voxel increases. The top row (a–c) shows mean FFA values with standard deviation (SD) over 30 realizations, enabling assessment of convergence and sensitivity to directional sampling under three Rician noise conditions: no noise, moderate (0.05), and high (0.09).

In the noise-free case, FFA remains constant across all sampling densities, showing no observable variation. Under moderate and high Rician noise, small fluctuations are observed at low numbers of directions, but beyond a moderate sampling density ($N \ge 500$), further increases produce minimal changes in FFA for all fiber configurations. While higher noise introduces additional variability, this variability appears largely attributable to measurement noise rather than directional discretization.

The relative ordering of diffusion environments is preserved across sampling densities and noise levels, indicating that FFA discriminates between fiber configurations. Although isotropic and three-fiber values are generally very close, a small separation is observable in the noise-free case, particularly for approximately 50 to 150 directions per voxel, suggesting an effective trade-off between sensitivity and computational cost.

The bottom row (d–f) quantifies convergence via relative error with respect to the high-density reference ($N=3000$). The relative error is calculated as:

$$\text {Relative error (\%)} = \frac{\left| \text {FFA}_{\text {mean}}(N) - \text {FFA}_{\text {mean}}(N=3000) \right| }{\left| \text {FFA}_{\text {mean}}(N=3000) \right| } \times 100$$

The error decreases monotonically with *N* and drops below the 1% threshold for $N \ge 1500$–2000 in isotropic and 3-fiber configurations, and much earlier ($N \ge 50$–200) in 1-fiber and 2-fiber configurations. This behavior is consistent across noise levels, though higher noise slightly delays full convergence in multi-fiber cases. These results show consistent convergence behavior and suggest that FFA becomes largely insensitive to the choice of evaluation directions once moderate sampling densities are reached.

Consistent with the observed reduction in relative error and the marked narrowing of the standard deviation, stable voxelwise FFA estimates are obtained at sufficiently high directional sampling densities, with 1-fiber and 2-fiber configurations reaching stability at substantially lower sampling levels.

#### Regularization effects on quartic tensor and Finsler metric estimation errors

To evaluate the impact of regularization on model estimation, we analyzed errors in both the quartic tensor and the derived Finsler metric under moderate Rician noise (0.05). Estimation accuracy was quantified using the relative root-mean-square error (RMSE), comparing unregularized fitting with Tikhonov-regularized fitting ($\lambda = 0.01$).

**Fig. 6:**
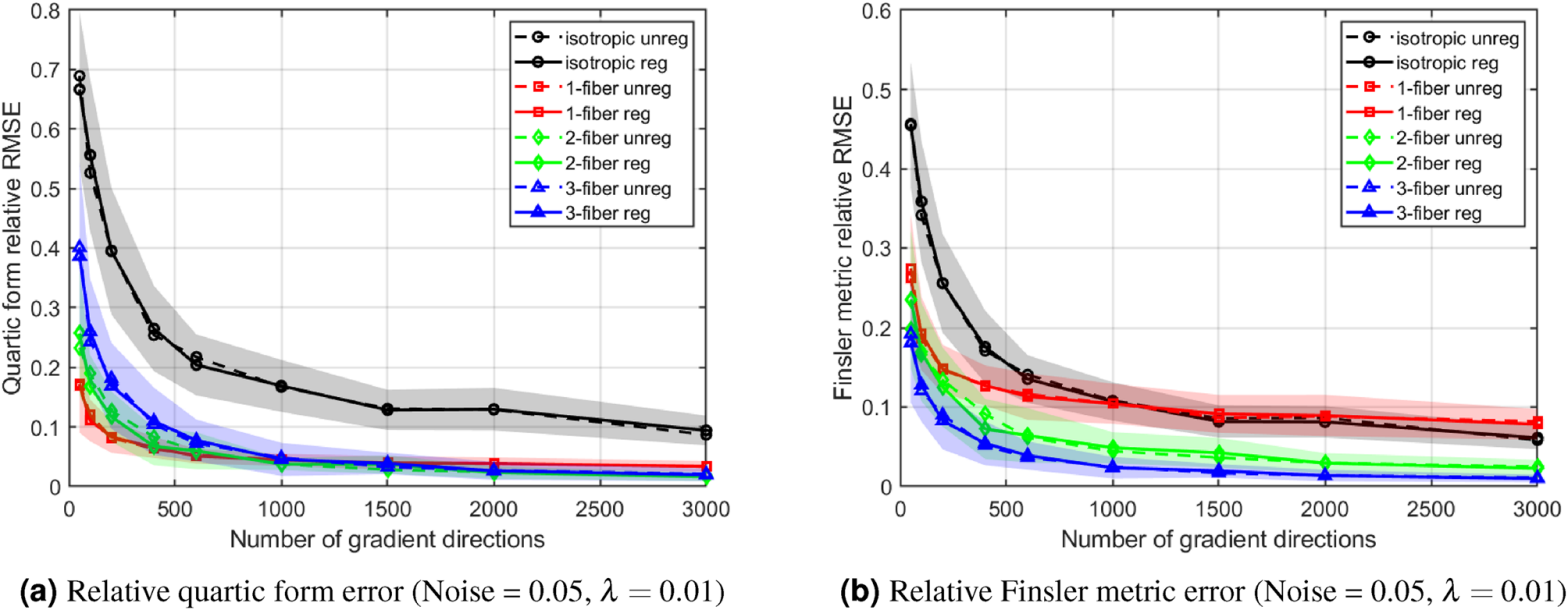
Effect of Tikhonov regularization on tensor estimation errors under moderate Rician noise (0.05). Left: Relative RMSE of the quartic form. Right: Relative RMSE of the Finsler metric. Solid lines/darker shading indicate regularized fitting ($\lambda = 0.01$), whereas dashed lines/lighter shading correspond to unregularized fitting ($\lambda = 0$). Curves show the mean ± standard deviation (shaded region) across 30 Monte Carlo realizations for different fiber configurations. Regularization consistently reduces error and variability, with the largest improvements observed in crossing-fiber cases (2- and 3-fiber) and at lower to moderate sampling densities. Errors converge to low values as the number of directions increases in both cases, although regularization enables reliable estimation with fewer measurements.

Figure 6 compares estimation errors obtained with and without regularization. Regularization consistently reduces both RMSE magnitude and variability, particularly in crossing-fiber configurations and at lower sampling densities. Errors decrease as the number of gradient directions increases in both cases, but regularization enables reliable estimation with fewer measurements.

The quartic form error serves as a direct measure of tensor fitting accuracy, whereas the Finsler metric provides a more application-relevant anisotropy index. Slightly larger errors for the Finsler metric are consistent with the propagation of small coefficient errors through its nonlinear formulation, which regularization mitigates. Overall, the primary reduction in noise sensitivity arises from directional averaging inherent in FFA computation and sufficient sampling density, while regularization provides additional stability at lower sampling densities and for 2-fiber and 3-fiber geometries.

These observations suggest that FFA stability aligns with underlying tensor convergence. A fixed, moderate number of gradient directions appears sufficient in these simulations to compute voxel-wise anisotropy reliably while preserving relative differences between diffusion configurations. While additional experiments with higher regularization ($\lambda = 0.05$) were performed and showed similar stabilizing trends, we present the results for $\lambda = 0.01$ as it provides an optimal balance between error reduction and preservation of the original tensor features.

Future studies incorporating non-orthogonal crossings, in vivo data, larger datasets, and more diverse conditions would be valuable to clarify these trends and support broader conclusions.

### Qualitative illustration of noise impact on tensor behavior and fiber classification

This section examines the effect of noise on the behavior of isotropic voxels and fiber classification. As noise levels increase, the signal profiles of anisotropic (especially three fibers per voxel) and isotropic voxels exhibit increasing similarity. This noise-induced ambiguity can lead to potential challenges in classification, where isotropic regions may appear to possess multi-fiber characteristics.

**Fig. 7:**
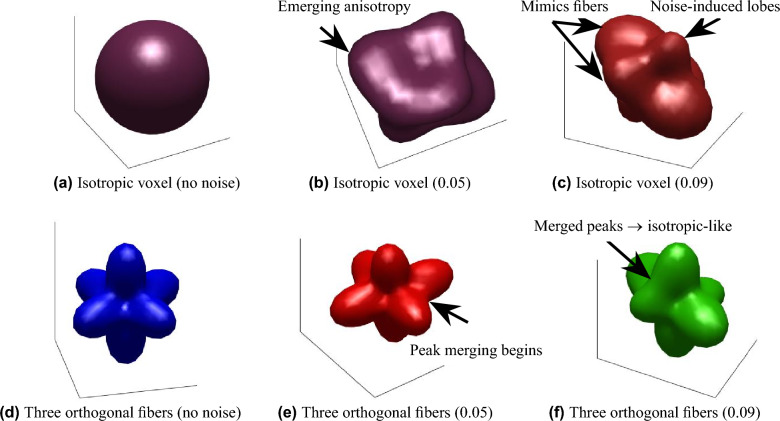
Tensor behavior under varying Rician noise levels. The first row shows isotropic voxels with increasing levels of noise: (**a**) no noise, (**b**) 0.05 noise, and (**c**) 0.09 noise. The second row illustrates three orthogonal fibers with the same Rician noise levels: (**d**) no noise, (**e**) 0.05 noise, and (**f**) 0.09 noise. Arrows indicate noise-induced lobes, peak merging, and features that appear morphologically similar to multi-fiber or isotropic configurations under noise (only a few representative examples are shown to avoid clutter). As noise increases, isotropic voxels begin to exhibit behavior resembling three fibers per voxel, and vice versa, highlighting the impact of noise on fiber classification.

As shown in Fig. 7, the first row illustrates isotropic voxels under varying Rician noise levels: with no noise (see Fig. 7a), with 0.05 noise (see Fig. 7b), and with 0.09 noise (see Fig. 7c). In the absence of noise, isotropic voxels appear homogeneous. However, as the noise level increases, the isotropic voxel behavior begins to display spurious directional features analogous to multi-fiber configurations.

The second row depicts three orthogonal fibers, selected for their high symmetry close to the isotropic case, under the same noise conditions. These are: with no noise (see Fig. 7d), with 0.05 noise (see Fig. 7e), and with 0.09 noise (see Fig. 7f). With no noise, the fibers remain distinguishable. However, as noise levels increase, the fibers become less distinct, complicating their classification. Under high noise, these three orthogonal fibers exhibit behavior that increasingly resembles isotropic voxels. This highlights the confounding effect of isotropic voxels when compared to the three-fiber case. The transition from isotropic to multi-fiber and vice versa emphasizes the challenges faced in practical diffusion imaging. It underscores the need for developing noise-resilient fiber classification methods and suggests that denoising techniques could help mitigate this effect.

### Evaluating descriptor performance on synthetic complex fiber arrangements

**Fig. 8:**
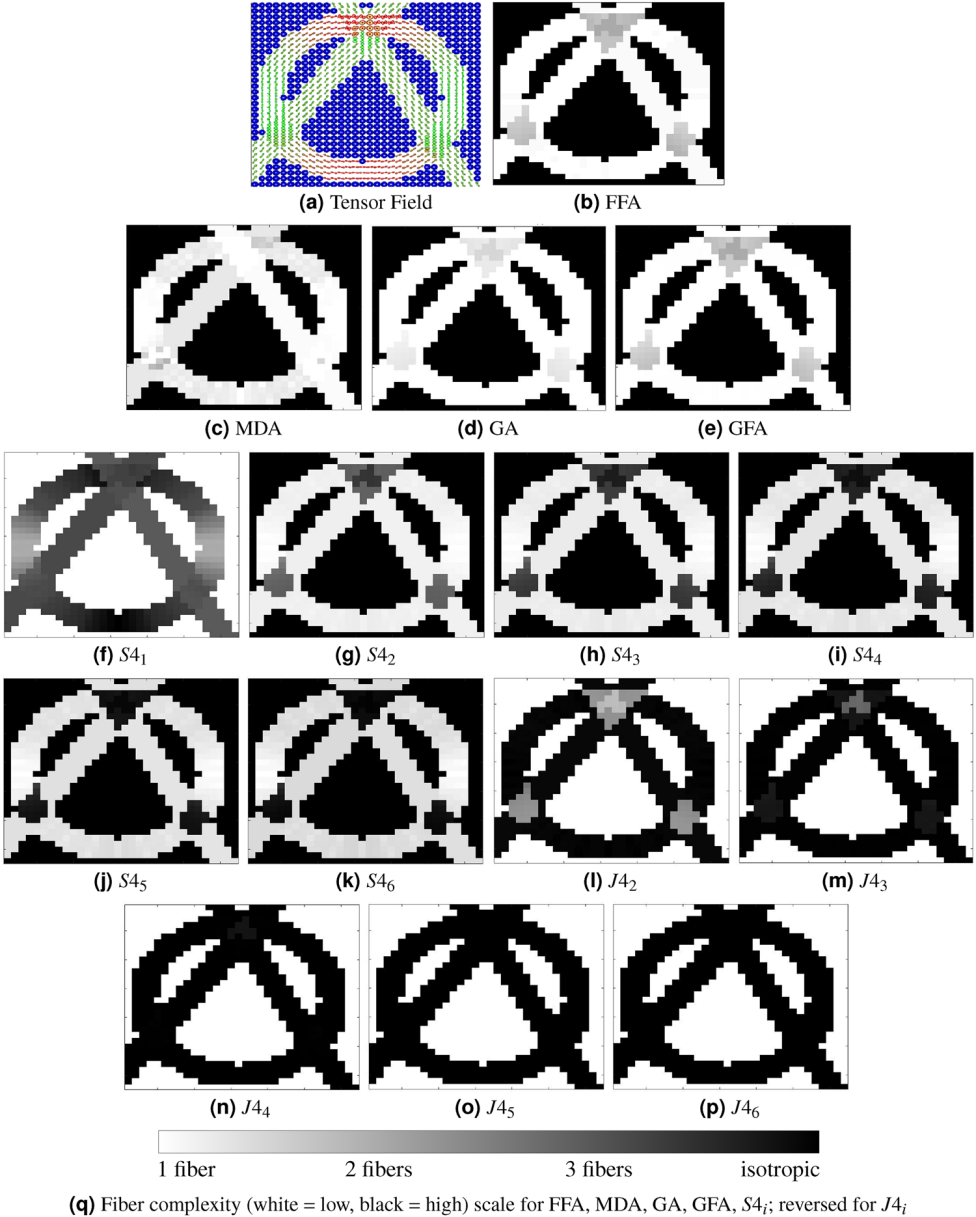
(**a**) Synthetic 4th-order tensor field showing regions with isotropic, single, double, and triple fiber configurations in the XY plane, created by varying crossing angles. (**b**) FFA map effectively differentiates these configurations, showing a clear contrast between the four regions. (**c**) MDA shows limited sensitivity to the underlying fiber configurations, resulting in reduced contrast. (**d**) GA exhibits lower contrast and is less effective at differentiating regions with two crossing fibers at small angles, whereas (**e**) GFA maintains contrast comparable to FFA. (**f**–**p**) Kelvin invariants ($S4_1$–$S4_6$ and $J4_2$–$J4_6$), displayed with 10th–90th percentile intensity windowing, show limited clarity in distinguishing fiber configurations. While $S4_2$ and $J4_2$ distinguish the four fiber categories in this synthetic example, their contrast is generally comparable to FFA. In contrast, $S4_3$ and $S4_4$ misrepresent the three-fiber region as isotropic, and $J4_4$ and $J4_5$ fail to distinguish single fibers from regions with two crossing fibers. These results indicate that FFA shows clearer separation of complex fiber arrangements compared to these scalar descriptors. (**g**) Relative fiber complexity scale bar for the XY plane.

We designed a complex fiber arrangement to evaluate the proposed scalar (see Fig. 8). The simulation used a tensor field with multiple crossing configurations (see Fig. 8a), combining two linear fibers and one circular fiber. This created four regions: isotropic diffusion (uniform pixels), one fiber (single dominant orientation), two fibers (crossing configuration), and three fibers (overlapping fibers with complex diffusion patterns). These regions, formed by intersections at varying angles from the circular fiber, are clearly visible. Unlike the previous experiment, the three-fiber configuration lies on the XY plane rather than a fully orthogonal configuration in three-dimensional space (XYZ plane).

- *FFA* The grayscale images in Fig. 8bshow the results of the proposed descriptor. It provides clear contrast between the four regions, isotropic, single fiber, two fibers, and three fibers, allowing each region to be visually distinguished.
- *MDA* As shown in Fig. 8c, MDA shows limited ability to classify the four regions. Even in the presence of two orthogonal crossing fibers, MDA values remain high and provide insufficient contrast to distinguish them^23^. Although a sliding window can reveal some contrast, it compresses values into a narrow range instead of spanning 0 to 1. Windowing also introduces challenges, such as selecting a consistent window size, limiting MDA’s reliability for analysis.
- *GA* As shown in Fig. 8d, GA shows lower contrast than FFA and cannot clearly differentiate all four regions. It struggles with crossing fibers at small angles, such as those from circular and linear configurations, often treating them as a single fiber and missing distinct contrast patterns.
- *GFA* As shown in Fig. 8e, GFA provides results comparable to FFA and effectively handles small crossing angles. It captures overall diffusion patterns and offers a reliable scalar measure of anisotropy, but it lacks detailed directional information, unlike the proposed descriptor, which can resolve multiple fiber orientations.
- *Kelvin invariants* The Kelvin scalars (see Fig. 8f–p) exhibit limited capacity to classify all four regions within this synthetic framework. Specifically, $S4_1$, $J4_4$, $J4_5$, and $J4_6$ demonstrate low sensitivity for fiber classification, while $S4_3$, $S4_4$, $S4_5$, and $S4_6$ do not provide sufficient contrast to differentiate between isotropic and three-fiber regions. Similarly, $J4_3$ shows reduced performance in distinguishing one- and two-fiber arrangements, particularly at small crossing angles where contrast is diminished. In contrast, $S4_2$ and $J4_2$ yield contrast levels comparable to FFA, although $J4_2$’s contrast is inverted. To facilitate visualization, each Kelvin invariant map was independently contrast-normalized using percentile-based intensity windowing (10th–90th percentiles) before display. Overall, these preliminary results suggest that FFA exhibits better performance in classifying complex fiber arrangements compared to most Kelvin invariants in this specific context. However, Kelvin invariants contain specialized information and are used in material science, mechanics, and biological applications. They describe the geometric properties and anisotropic behavior of materials under deformation. Despite their value in these fields, they are less effective for classifying white matter fibers, as observed in our simulations. This is because they have a limited ability to capture the complex fiber arrangements found in the brain.

### Directional sensitivity of FFA maps in synthetic data

**Fig. 9:**
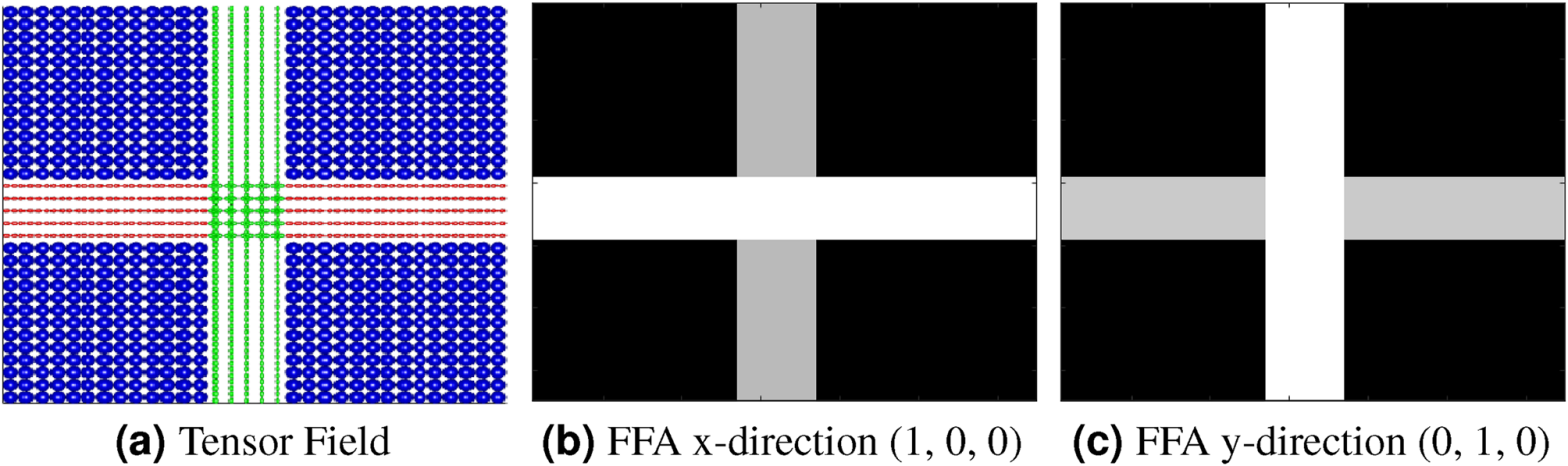
(**a**) Synthetic 4th-order tensor field. (**b**) FFA map computed using a subset of gradient directions consisting of a single vector pointing along the $x$-axis $(1, 0, 0)$; estimated diffusion directions are shown in white. (**c**) Similarly, the FFA map computed along the $y$-axis $(0, 1, 0)$; estimated diffusion directions are shown in white. These FFA maps highlight fiber directionality by isolating contributions from specific structural orientations.

The directional sensitivity of FFA maps was examined using a simplified synthetic experiment. Figure 9 demonstrates how subsets of gradient directions can be used in FFA calculations to probe specific structural orientations. Here, for illustrative purposes, we show results using a single gradient direction: Fig. 9b,c display FFA maps computed using a gradient pointing along the x-direction (1, 0, 0) and y-direction (0, 1, 0), respectively. In these maps, areas of high directional diffusion are highlighted in white, reflecting the alignment with the specific gradient orientation. This experiment shows that FFA’s sensitivity to directional gradients helps differentiate overlapping fibers and allows better characterization of complex fiber arrangements within a voxel, an area that remains challenging for conventional approaches.

By capturing subtle changes in diffusion profiles based on gradient direction, FFA may provide detailed insights into fiber orientation and structure within a voxel. This capability is potentially beneficial in white matter regions, where densely packed fibers with small angular separations pose significant challenges to accurate classification^33–35^.

Furthermore, FFA leverages subsets of gradient directions to not only quantify the number of fibers but also reveal their directional orientation. This ability to provide both quantitative and directional information offers a distinct advantage over standard diffusion models.

### Evaluating descriptor performance on in vivo data

**Fig. 10:**
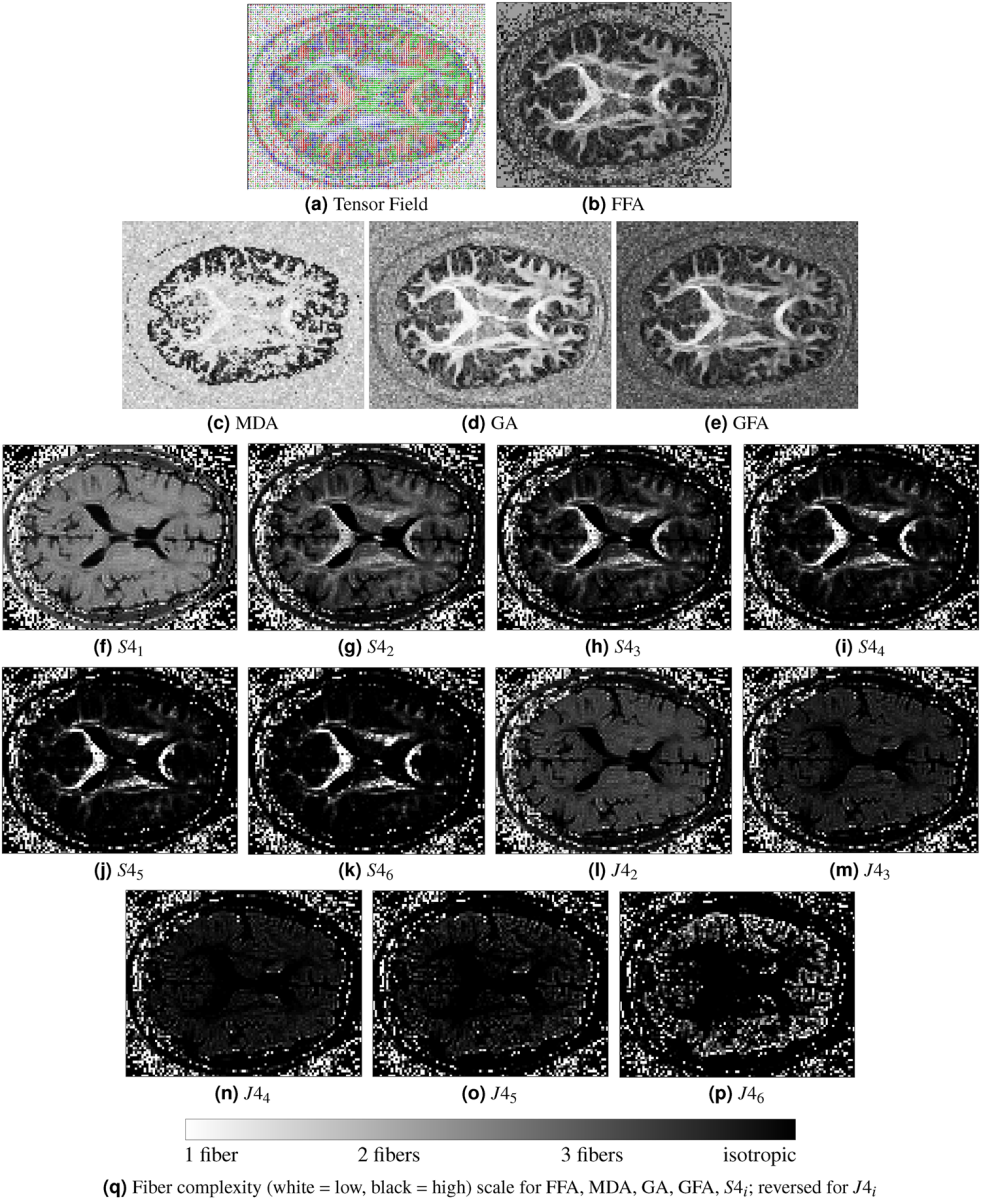
Comparison of FFA with existing scalars on an in vivo brain dataset (axial slice). (**a**) The 4th-order tensor field shows fiber orientations, with each voxel representing the primary fiber direction. (**b**) FFA highlights high anisotropy regions, revealing tissue structure contrast and orientation. (**c**) MDA and (**d**) GA provide lower contrast, while (**e**) GFA performs comparably to FFA. (**f**–**p**) Kelvin invariants: from $S4_1$ to $S4_6$ and from $J4_2$ to $J4_6$ capture varying detail levels. Kelvin maps are rescaled using intensity windowing (10th–90th percentiles) for contrast enhancement, while other maps are shown on their original scale. FFA provides improved tissue differentiation relative to existing scalars. (**g**) Relative fiber complexity scale bar for the XYZ plane.

We evaluated the proposed descriptor using in vivo diffusion-weighted MRI data (axial slice) and compared it with existing scalars (see Fig. 10). The corresponding tensor field is shown in Fig. 10a. Key observations from these figures include:

- *FFA* This descriptor shows clear contrast across the brain in the examined axial slice (see Fig. 10b), distinguishing white and gray matter and highlighting structural features. These findings suggest potential for in vivo applications, warranting further validation in multi-slice and multi-subject studies.
- *MDA, GA, and GFA* MDA (see Fig. 10c) produces high anisotropy values, even in regions with crossing fibers, thereby reducing interpretability. GA (see Fig. 10d) exhibits lower contrast and struggles with small crossing angles, as elevated anisotropy values reduce the contrast between fiber configurations. GFA (see Fig. 10e) performs comparably to FFA and may serve as a simpler alternative in some scenarios. In the above cases, all results are shown on their original scale [0, 1], without windowing.
- *Kelvin invariants* Maps from $S4_1$ to $J4_6$ (see Fig. 10f–p) provide variable contrast. While some regions are visually clear, these scalars fail to retain crucial structural details and are limited for fiber classification. For visualization, each Kelvin invariant map was independently contrast-normalized using 10th–90th percentile windowing, enhancing display contrast but limiting direct interpretation of absolute values. Scalars like $J4_4$, $J4_5$, and $J4_6$ miss key tissue features, and even $J4_2$ and $S4_2$, which performed well in simulations, offer limited contrast in vivo due to noise, partial volume effects, and biological variability. These observations suggest that FFA maintains clearer structural detail under the conditions shown.

Overall, Fig. 10 suggests that FFA shows clearer structural detail and more consistent contrast compared to Kelvin invariants and other scalars in the displayed axial slice. These initial in vivo observations are encouraging but remain preliminary.

### Directional sensitivity of FFA maps in in vivo data

**Fig. 11:**
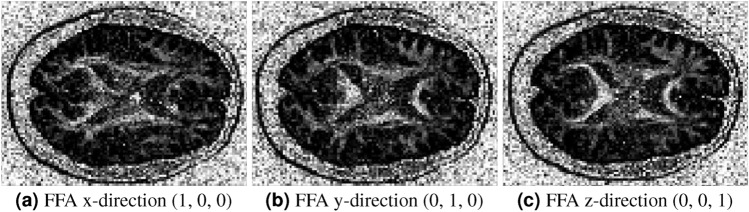
FFA maps from an axial slice of in vivo brain data illustrate their sensitivity to different gradient orientations, effectively capturing direction-specific diffusion contrasts. The corresponding diffusion directions, indicated in white on the maps (as in Fig. 9), highlight FFA’s capability to characterize fiber orientation and underlying tissue structure. Such maps can be generated for any set of gradient orientations, providing a flexible tool for diffusion analysis.

Figure 11 shows FFA maps for an in vivo brain axial slice, computed along each of the primary gradient directions (x: 1,0,0; y: 0,1,0; z: 0,0,1). These maps highlight FFA’s sensitivity to diffusion directionality, with each map emphasizing features specific to its gradient orientation. Examining multiple directional maps can help reveal features of fiber orientation and tissue structure, providing illustrative insight into diffusion patterns. Diffusion directions are indicated in white (as in Fig. 9), reflecting FFA’s ability to characterize complex fiber arrangements. This approach offers a flexible and informative tool for in vivo diffusion analysis.

### Classification ranges and their practical implications

For classifying tissue types using the proposed descriptor, it is essential to select appropriate and well-justified ranges of descriptor values. Ideally, these ranges should be informed by both synthetic and in vivo data, as well as anatomical knowledge of white matter fiber organization.

**Table 3:** Proposed descriptor value ranges used to classify voxels based on the expected number of fibers with distinct orientations.

Number of fibers per voxel	Classification range
One	[0.70, 1.00]
Two	[0.40, 0.70)
Three	[0.20, 0.40)
Isotropic	[0.00, 0.20)

Table 3 provides suggested classification ranges, primarily guided by synthetic data analysis (see Fig. 2). The synthetic results serve as a reference for interpreting in vivo data and understanding how descriptor values may correspond to fiber configurations.

**Fig. 12:**
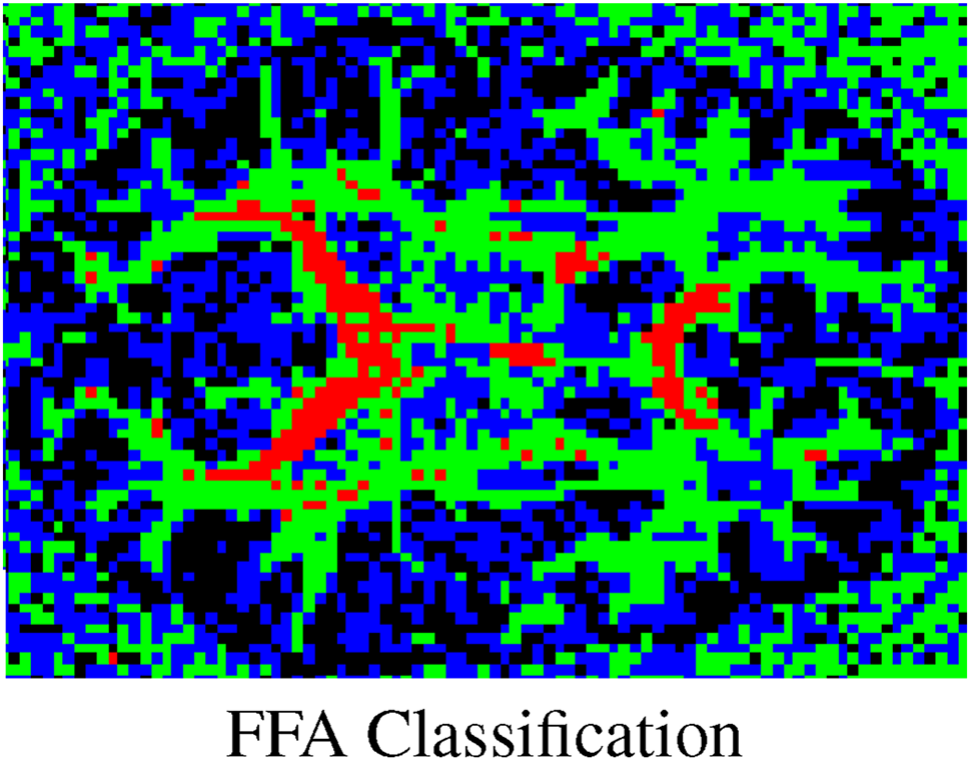
Classification maps of an in vivo brain axial slice using the proposed descriptor. Color coding indicates fiber orientations: red for one fiber, green for two fibers, blue for three fibers, and black for isotropic regions. The corresponding voxel ranges are detailed in Table 3.

Figure 12 demonstrates the practical application of these ranges to in vivo data. Voxels are color-coded to indicate fiber configurations: red for one fiber, green for two fibers, blue for three fibers, and black for isotropic regions. These maps provide an initial insight into fiber arrangements that generally align with known brain anatomy.

These classifications illustrate how the proposed descriptor distinguishes different tissue types. Validation against anatomical data is crucial, particularly because real images contain noise, which complicates the precise determination of classification ranges. The ranges in this study were derived from synthetic data with an estimated Rician noise level of  0.05, based on visual inspection of the data distribution.

Despite these challenges, the results highlight the potential of the proposed descriptor for differentiating fiber configurations in vivo. The three-fiber-per-voxel configuration is especially challenging, as it can resemble isotropic voxels (see Fig. 7). Emphasizing cases of three orthogonal crossings provides a proof-of-concept, demonstrating the preliminary feasibility and initial effectiveness of the descriptor in characterizing simulated fiber configurations and observed diffusion patterns.

## Discussion

The results of both statistical significance testing and visual evaluation indicate that the proposed Finsler fractional anisotropy (FFA) differentiates between isotropic regions and voxels with distinct fiber orientations in the tested conditions. These preliminary findings suggest its potential as a descriptor for diffusion imaging, particularly for exploring the classification of white matter microstructure. Compared with conventional anisotropy descriptors, FFA shows improved sensitivity to underlying fiber geometry and better handling of directionally complex multi-fiber arrangements in both synthetic simulations and the proof-of-concept in vivo evaluation presented here.

At the same time, several limitations must be considered. FFA shows sensitivity to noise, particularly in datasets with low signal-to-noise ratios (SNR). This sensitivity may lead to reduced precision and less smooth contrast in diffusion maps. As suggested by our stability analysis, regularization strategies, such as Tikhonov regularization, can help mitigate these effects by stabilizing the tensor fields while preserving essential anatomical features. While we utilized a computationally efficient baseline here, other approaches, such as spatial regularization, variational methods, or total variation (TV) minimization, along with multi-acquisition averaging or advanced denoising techniques, could further help mitigate these effects by preserving underlying anatomical features while reducing fluctuations in the estimated fields.

The choice of a 4th-order diffusion tensor for defining FFA reflects a strategic balance between descriptive capability, noise robustness, and computational feasibility. While higher-order tensors (e.g., 6th or 8th order) offer greater expressive power, their high dimensionality (28 or 45 independent components, respectively) increases sensitivity to noise and estimation instability under standard in vivo angular sampling. Furthermore, because most brain voxels contain only a few dominant fiber populations, a 4th-order tensor provides sufficient flexibility to capture biologically meaningful complexity without over-parameterizing the model. We acknowledge that exploring higher-order tensors remains an interesting direction for future work as acquisition techniques and SNR continue to improve. Further testing across diverse acquisition protocols, multiple subjects, and clinical populations will be essential to evaluate the performance of FFA under varied real-world conditions.

So far, the Finsler framework has been primarily applied to tractography, and no scalar biomarker has previously been developed within this space. Our work, therefore, represents an initial step toward filling this gap by extending Finsler-based modeling to the definition of a scalar anisotropy descriptor. This transition broadens the scope of Finsler methods from trajectory reconstruction to quantitative tissue characterization, potentially opening avenues for new biomarkers of microstructural integrity.

Future research should focus on refining the classification capabilities of FFA. Key priorities include optimizing thresholds for multi-fiber configurations, enhancing noise resilience in in vivo data, and benchmarking against histological or high-resolution anatomical references. Additionally, adopting a spherical-weighted averaging approach, exploring alternative aggregation methods, and improving computational efficiency will also help explore FFA as a potential tool in clinical and research settings. By addressing these challenges, FFA may contribute to the analysis of complex white matter structures and support improved diagnostic and monitoring strategies in neurological disorders.

In conclusion, FFA offers promising properties in diffusion tensor imaging. Leveraging nonlinear Finsler geometry, it provides detailed and directionally sensitive insights into complex white matter structures in the evaluated data, including the ability to probe orientation-specific anisotropy through subsets of gradient directions, thereby potentially addressing some limitations associated with traditional scalar descriptors. The observed performance of this metric across the tested datasets and experimental conditions suggests possible utility for further investigation in neurological conditions such as multiple sclerosis and Alzheimer’s disease. FFA could therefore serve as a candidate complementary tool in the ongoing development of diffusion imaging techniques, pending further validation in larger and more diverse cohorts.

## Methods

Finsler geometry generalizes Riemannian geometry by incorporating both position and direction^33^, making it well-suited for modeling highly anisotropic diffusion in brain tissue. Leveraging this framework allows us to capture multiple fiber populations within a single voxel, providing enhanced sensitivity to complex fiber arrangements compared with conventional tensor-based methods.

### Finsler fractional anisotropy (FFA)

The Finsler norm $F(\vec {x}, \vec {y})$ quantifies the effective diffusion resistance along a direction $\vec {y}$ at position $\vec {x}$. Using Einstein summation, this norm is given by the positive fourth root of a homogeneous quartic form defined by the fourth-order diffusion tensor:

1 $$\begin{aligned} F(\vec {x}, \vec {y}) = \left( D_4(\vec {x}, \vec {y}) \right) ^{1/4}, \quad D_4(\vec {x}, \vec {y}) = D_{ijkl}(\vec {x}) y^i y^j y^k y^l, \end{aligned}$$

where $D_{ijkl}(\vec {x})$ are the components of the 4th-order diffusion tensor. From this norm, a direction-dependent (local) Finsler metric tensor is derived:

2 $$\begin{aligned} g_{ij}(\vec {x}, \vec {y}) = \frac{1}{2} \frac{\partial ^2 F^2(\vec {x}, \vec {y})}{\partial y^i \partial y^j}. \end{aligned}$$

FFA is computed at each voxel using the following procedure:

1. *Directional sampling* Define a set of unit vectors $\{\vec {v}_s\}_{s=1}^p$, sampled uniformly on the unit sphere (or, exploiting antipodal symmetry of diffusion MRI, on a hemisphere, since opposite gradient directions yield identical signal contributions).
2. *Local metric derivation* For each sampled direction $\vec {v}_s$, the corresponding local Finsler metric tensor $g_{ij}(\vec {x}, \vec {v}_s)$ is calculated using Eq. (2).
3. *Metric averaging* These local tensors are averaged across selected sampled orientations to obtain a mean metric tensor, which encapsulates the underlying microstructural complexity:
  3 $$\begin{aligned} \bar{g}_{ij}(\vec {x}) = \frac{1}{p} \sum _{s=1}^p g_{ij}(\vec {x}, \vec {v}_s). \end{aligned}$$
4. *Anisotropy mapping* Finally, the FFA is determined by applying the standard fractional anisotropy formula to the eigenvalues of this mean metric tensor:
  4 $$\begin{aligned} \text {FFA}(\vec {x}) = \text {FA} \left( \bar{g}_{ij}(\vec {x}) \right) . \end{aligned}$$

Because FFA depends on the chosen set of gradient directions, it can be used to explore different aspects of the diffusion profile, potentially offering improved sensitivity in regions with crossing fibers or complex microstructure.

### Synthetic data generation

Synthetic diffusion-weighted images were generated using MATLAB fanDTasia ToolBox^37,38^ to simulate water diffusion along white matter fibers. Synthetic datasets provide controlled conditions for evaluating descriptor performance with known fiber configurations.

To simulate realistic MRI acquisition conditions, Rician noise was added to the synthetic diffusion-weighted signals. Complex Gaussian noise with zero mean and standard deviation $\sigma$ was independently added to the real and imaginary components, and the magnitude signal was then computed, resulting in Rician-distributed noise. The signal-to-noise ratio (SNR) was defined as

5 $$\begin{aligned} \text {SNR} = \frac{S_0}{\sigma }, \end{aligned}$$

where $S_0$ denotes the non-diffusion-weighted baseline signal ($b=0$). For all synthetic experiments, $S_0$ was normalized to 1, and noise levels $\sigma \in \{0.00, 0.01, 0.05, 0.09\}$ correspond to SNR values of $\infty$, 100, 20, and $\approx 11$, simulating a range from ideal to clinical-grade data.

Key parameters for the synthetic datasets are summarized in Table 4.

**Table 4:** Summary of key experimental parameters for synthetic datasets.

Parameter	Synthetic dataset
*b*-value ($s/mm^{2}$)	1500
Number of gradient directions	81
Axial diffusivity, $\lambda _1$ (mm$^2$/s)	$1.7 \times 10^{-3}$
Radial diffusivities, $\lambda _2$, $\lambda _3$ (mm$^2$/s)	$0.2 \times 10^{-3}$
Subject	Synthetic white matter simulation
Software	MATLAB fanDTasia ToolBox (for simulation data generation)
Processing hardware	Intel(R) Core(TM) i5-7500T CPU, 16GB RAM
Software version	MATLAB 2019a
Rician noise levels ($\sigma$)	$$\begin{array}{llll} 0&0.01&0.05&0.09 \end{array}$$
Corresponding SNR values ($S_0 = 1$)	$$\begin{array}{llll} \infty&100&20&\hspace{5pt}\approx 11 \end{array}$$

### In vivo data acquisition

In vivo diffusion-weighted imaging (DWI) data were collected from a healthy volunteer using a Siemens MAGNETOM Prisma 3T MRI scanner at CEITEC, Masaryk University, Brno, Czech Republic. These datasets serve to validate the descriptor under real brain conditions, accounting for biological variability, partial volume effects, and noise.

Key acquisition parameters are summarized in Table 5.

**Table 5:** Summary of key experimental parameters for in vivo DWI dataset.

Parameter	In vivo dataset (Siemens 3T MRI)
*b*-value ($s/mm^{2}$)	1000
Number of gradient directions	64
Repetition time (TR)	4000 ms
Echo time (TE)	85 ms
Bandwidth	1560 Hz/Px
Slice thickness	2 mm
Field of view (FoV)	$230 \times 230 \times 150$ mm
Voxel resolution	$2 \times 2 \times 2$ mm$^3$ (isotropic)
Reconstructed image dimensions	$114 \times 114 \times 70$ voxels
Number of slices	70
Partial Fourier	6/8 (75%)
Fat suppression	Spectral adiabatic inversion recovery (SPAIR)
Parallel imaging technique	GeneRalized autocalibrating partially parallel acquisitions (GRAPPA)
Acceleration factor	2
Scan time	7 min
Scan type	Diffusion weighted imaging (DWI)
Scanner model	Siemens MAGNETOM Prisma 3T
Acquisition protocol	Siemens EPI-based DWI protocol
Gradient system	XR gradients (max strength: 80 mT/m, Max Slew Rate: 200 T/m/s)
Software version	Siemens syngo MR XA30
Magnet homogeneity	0.2 ppm within 40 cm DSV
Reconstruction and post-processing	Siemens syngo.via
Processing hardware	Intel(R) Core(TM) i7-9700K CPU, 32GB RAM
Subject	Healthy volunteer (Brno, Czech Republic)

## Data Availability

The anonymized in vivo diffusion-weighted MRI datasets used in this study are available from the corresponding author upon reasonable request. Data sharing complies with the original informed consent and ethical approval (Ref. No. EKV-2018-010).
